# Crystal Structure of Dimeric Flavodoxin from *Desulfovibrio gigas* Suggests a Potential Binding Region for the Electron-Transferring Partner

**DOI:** 10.3390/ijms14011667

**Published:** 2013-01-15

**Authors:** Yin-Cheng Hsieh, Tze Shyang Chia, Hoong-Kun Fun, Chun-Jung Chen

**Affiliations:** 1 Life Science Group, Scientific Research Division, National Synchrotron Radiation Research Center, Hsinchu 30076, Taiwan; E-Mail: yinchengh@gmail.com; 2 X-ray Crystallography Unit, School of Physics, Universiti Sains Malaysia, 11800 USM, Penang, Malaysia; E-Mails: chiatzeshyang@hotmail.com (T.S.C.); hkfun@usm.my (H.-K.F.); 3 Department of Pharmaceutical Chemistry, College of Pharmacy, King Saud University, Riyadh 11451, Saudi Arabia; E-Mail: hfun.c@ksu.edu.sa; 4 Department of Physics, National Tsing Hua University, Hsinchu 30043, Taiwan; 5 Institute of Biotechnology, National Cheng Kung University, Tainan City 70101, Taiwan; 6 University Center for Bioscience and Biotechnology, National Cheng Kung University, Tainan City 70101, Taiwan

**Keywords:** flavodoxin (Fld), flavin mononucleotide (FMN), crystal structure, dimer, binding region

## Abstract

Flavodoxins, which exist widely in microorganisms, have been found in various pathways with multiple physiological functions. The flavodoxin (Fld) containing the cofactor flavin mononucleotide (FMN) from sulfur-reducing bacteria *Desulfovibrio gigas* (*D. gigas*) is a short-chain enzyme that comprises 146 residues with a molecular mass of 15 kDa and plays important roles in the electron-transfer chain. To investigate its structure, we purified this Fld directly from anaerobically grown *D. gigas* cells. The crystal structure of Fld, determined at resolution 1.3 Å, is a dimer with two FMN packing in an orientation head to head at a distance of 17 Å, which generates a long and connected negatively charged region. Two loops, Thr59–Asp63 and Asp95–Tyr100, are located in the negatively charged region and between two FMN, and are structurally dynamic. An analysis of each monomer shows that the structure of Fld is in a semiquinone state; the positions of FMN and the surrounding residues in the active site deviate. The crystal structure of Fld from *D. gigas* agrees with a dimeric form in the solution state. The dimerization area, dynamic characteristics and structure variations between monomers enable us to identify a possible binding area for its functional partners.

## 1. Introduction

The electron-transfer chains are the critical reactions, such as the photosynthesis and respiration systems, for the production of biological energy. The chain reactions require several proteins, such as electron carriers, which transfer electrons through the pathways with the various co-factors, such as heme, the iron-cluster and flavin mononucleotide (FMN). Flavodoxins (Flds), which are electron-carrier flavoproteins of small molecular mass that contain FMN as the prosthetic group, are found in various microorganisms [1], and play multiple roles in varied pathways. For instance, Fld from *Anabaena* PCC 7119 and *Synechococcus* sp. 2 PCC 700 shuttle electrons from the PS-I (photosystem I) to FNR (ferredoxin-NADP^+^ reductase) [2–4] in photosynthetic reactions. Fld was shown to participate in the synthesis of methionine [5,6], HMBPP [7] and biotin [8]. In *Azotobacter vinelandii* and *Azotobacter chroococcum*, the Flds are related to nitrate reduction [9] and nitrogenase recognition [10], respectively.

Flds are reported to activate pyruvate-formate lyase [11] and ribonuclectide reductase [12]. Flds generally contain one non-covalently bound FMN that acts as an electron-transfer center by switching three redox states, including the hydroquinone, semiquinone and oxidized states. The lowest redox potentials of Flds are found in the range from −305 to −520 mV [13–15]. The reduction of the oxidized Fld to the semiquinone form is reported as easily achieved on adding chemical reducing agents, such as β-mercaptoethanol [14], whereas the reduction of semiquinone to hydroquinone form is relatively difficult because of the low redox. The physiological importance of Flds arises from their involvement in transitions between one- and two-electron reduced states. Preceding authors reported that the oxidized state might not be involved in the physiologically relevant redox reactions [16].

Sulfate-reducing bacteria (SRB) constitutes prokaryotes of a particular group that possess the capacity to metabolize sulfate. These bacteria are strict anaerobes, but are widespread in various anaerobic environments, such as soil, sediments, oil fields and the sea**,** and are found internally in animals, including human beings [17]. SRB also causes serious economic problems such as biocorrosion in the oil industry and souring of oil or gas deposits due to sulfide production [18,19]; however, their capacity to degrade sulfate is useful to prevent environmental pollution, as SRB can remove sulfate and toxic heavy atoms from factory waste waters [20]. *Desulfovibrio* is the most studied representative of SRB, making it an excellent model for the investigation of the respiration electron-transfer chain [21,22].

**Scheme 1:**
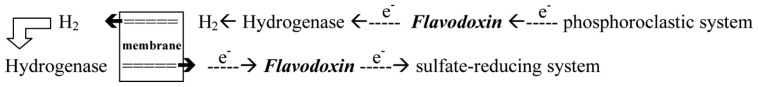
Proposed electron transfer chain in *Desulfovibrio* sp.

Flds in *Desulfovibrio* sp. are suggested to participate in two major reactions as shown in the scheme below (Scheme 1); the dashed lines represent other proteins participating in the reactions.

1. With the donation of electrons from the pyruvate, Fld can transport electrons from the phosphoroclastic system to the outer membrane or to the sulfate-reducing system [23]. The other organic source, aldehyde, which is reduced by aldehyde oxidoreductase (AOR), can also produce electrons. The transfer of electrons between AOR and Fld has also been reported [24].
2. Through the donation of electrons from molecular dihydrogen, Fld carries the electrons from the outer membrane to the sulfate-reducing system [25]. Although some studies showed that cytochrome c3 and Fld might form a protein complex [26], the exact pathway of the electron transfer between the periplasm and the sulfate-reducing system remains unclear. Among the SRB *Desulfovibrio* sp., the structures of Flds from *Desulfovibrio vulgaris* (*D. vulgaris*) and *Desulfovibrio desulfuricans* (*D. desulfuricans*) have been determined [27,28]. The effects of some mutated residues, such as G61V and D95E, interacting with the FMN in three redox states were also studied in *D. vulgaris* Fld [29,30].

Based on their molecular mass, the Flds are classifiable into two groups: the “long-chain” Flds containing about 169–176 amino acids and the “short-chain” enzymes lacking ~20–30 residues at the middle region of the sequences in comparison between these two groups, of which the physiological function is unknown. In the present work, we isolated a native functional “short-chain” Fld containing 146 amino acids with the cofactor FMN directly from anaerobically grown *D. gigas* cells. The Fld from *D. gigas* exhibited the typical UV and visible spectra at wavelengths 374 and 457 nm and showed that the sequence identities were less than 60% as related to enzymes from *D. vulgaris* and *D. desulfuricans* (Figure S1). We report here the structure of the short-chain Fld from *D. gigas* at a resolution of 1.3 Å; we study its structural-functional relationship and the prospective binding region for an electron-transferring partner.

## 2. Results and Discussion

### 2.1. Crystal Characterization and X-ray Diffraction

**Figure 1:**
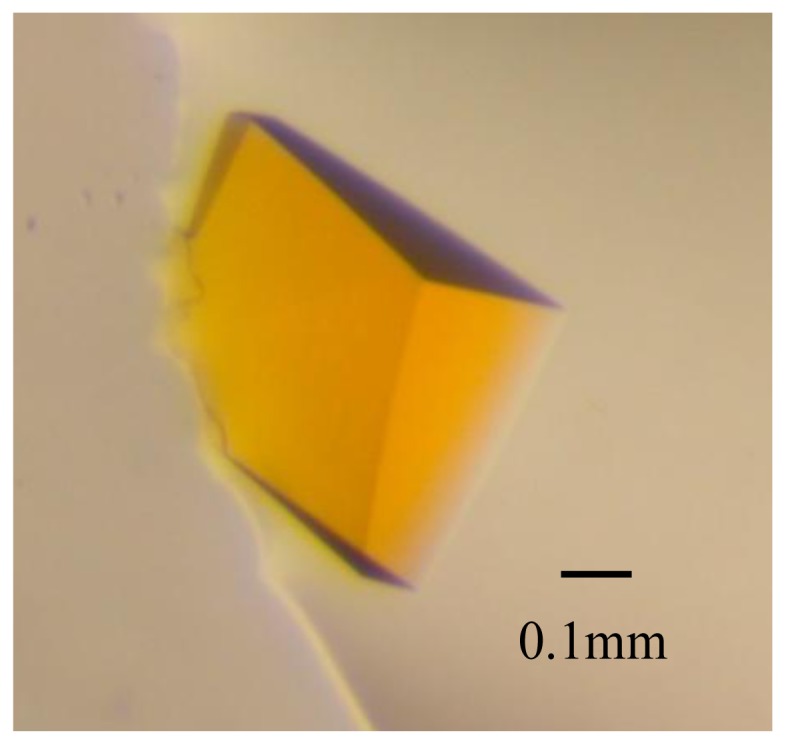
The crystal of Fld. The single yellow crystal was obtained after modification from the initial screening conditions.

Using a micro-seeding method, protein crystals of the rectangular shape appeared after two days, and continued to grow to a terminal size 0.5 × 0.4 × 0.4 mm^3^ within one week in an incubator at 18 °C (Figure 1). These crystals were sensitive to a variation of precipitant concentration while being transferred to the cryo-protectent solution containing glycerol (20%). Several crystals were tested before data collection because of a mosaicity > 1° that caused an overlap of diffraction spots in the high-resolution regions. Radiation damage was observed on protracted exposure during data collection, which caused *I*/σ(*I*) to decrease and *R*_sym_ to increase. Assuming the presence of two Fld molecules per asymmetric unit, the Matthew’s coefficient is estimated to be 2.06 Å^3^ Da^−1^, corresponding to a solvent content of 40.4% [31], which is within the normal range for protein crystals. A calculation of the self-rotation function showed extra peaks in addition to the crystallographic orthorhombic symmetry in κ = 180°, indicating that the non-crystallographic two-fold axes exist in the local symmetry of Fld molecules, which confirms the dimeric structure of Fld. Details of data statistics are summarized in Table 1.

### 2.2. The Crystal Structure of Fld

Although the crystal of Fld diffracted to resolution ~1.2 Å with completeness 99.7%, the data in the resolution shell 1.2–1.3 Å with a high *R*_sym_ (~70%) was unusable in the structural refinement; we therefore performed the refinement in a resolution range 30–1.3 Å. The refined structural model of Fld at resolution 1.3 Å gave factors *R_work_* = 18% and *R*_free_ = 21%. According to Ramachandran plot, the stereochemistry showed that 282 amino acids are in the most favored region and four residues in the allowed region, indicating a satisfactory structural refinement of dimeric Fld.

**Figure 2:**
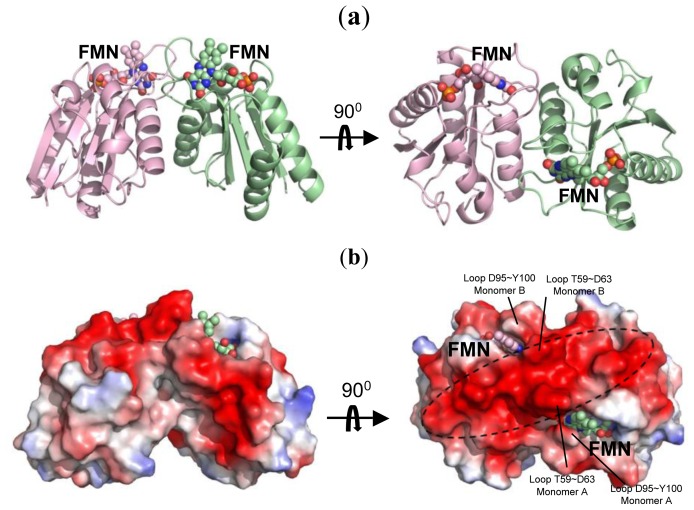
The overall structure of Fld from *D. gigas*. (**a**) The dimeric structure of Fld. The co-factor, flavin mononucleotide (FMN), is showed in sphere; (**b**) The electrostatic surface shows a wide area containing negative charges (red). In the right panel, the long and connected, negatively charged, region formed by dimerization of Fld is labeled with a dash circle. The dynamic loops T59–D63 and D95–Y100 from each monomer are labeled.

The overall structure of the monomer Fld, consistent of 145 amino acids (2–146), comprises five β-helices and five β-sheets in order ββββββββββ folding into a sandwich conformation equipping five β-sheets in the middle with two and three β-helices surrounding on both sides (Figure 2a). The co-factor FMN is clearly visible with the electron density, and is located near the protein surface. The framework of hydrogen bonds involving main-chain atoms, side-chain atoms and various water molecules, contributes greatly to the stability of FMN. The dimensions of dimeric Fld are ~59 × 39 × 35 Å^3^, with a surface area 3644 Å^2^ accessible to solvents. An inspection of the electrostatic surface shows that the negative charges distribute almost all around the protein surface because Fld has a small pI value (~4.08). The amino-acid composition of Fld contains large proportions of Asp (8.9%) and Glu (10.3%), which contribute the major negative charges on the protein surface. Some residues with polar side chains, such as Ser (5.5%) and Thr (6.8%), also provide negative charges on the surface. The Fld dimer forms a long and connected, negatively charged, surface with Asp40, Asp62, Asp63, Glu64, Glu66, Gln68, Glu69, Asp70, Tyr75, Glu76, Asp77, Thr99 and Tyr100, and with FMN located at both sides (Figure 2b).

### 2.3. Environment of FMN

**Figure 3:**
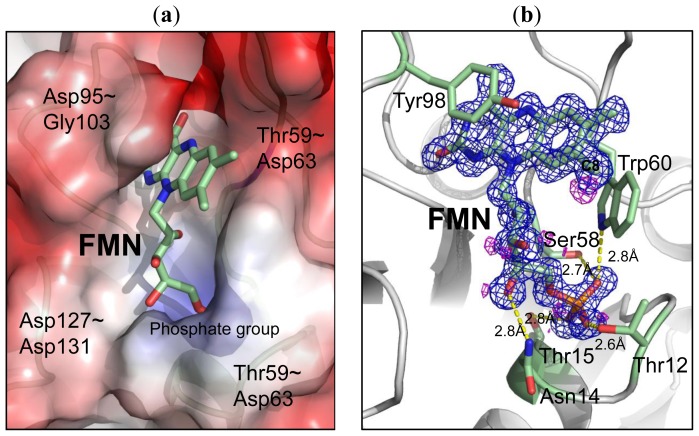
The co-factor FMN. (**a**) The electrostatic surface shows the charge distribution of the FMN-binding site with the displayed potentials range from −107 (red) to 107 (blue) kTe^−1^. Four loops involved in the formation of the binding pocket are labeled; (**b**) The electron density (composite-omit map with 2*F*_o_–*F*_c_, contour level = 1.2 σ, blue) of FMN reveals a high quality of the refined structure with a density hole of the isoalloxazine ring. The major interactions between residues and the FMN are labeled with dash lines (yellow). The *Fo–Fc* difference map (magenta, contour level = 2.5σ) showed only an extra electron density near C8 of FMN.

In the high resolution Fld structure (1.3 Å), the position of FMN was well defined by the electron density, resolving a hole of the aromatic ring (Figure 3). The Fld exposes its co-factor FMN to the surface with an accessible surface area ~32.5 Å^2^. The structure of FMN comprises two major parts; the “head” part with the isoalloxazine ring is more hydrophobic, whereas the “tail” part with the phosphate group is more hydrophilic. The electrostatic surface of the FMN-binding pocket reveals that the binding site for FMN is hydrophobic with aromatic residues, e.g., Trp60 and Phe101, whereas the binding pocket for the phosphate group is positively charged (Figure 3a). Four major loops, including Ser10–Thr15, Asp95–Gly103, Thr59–Asp63 and Asp127–Asp131, contribute to the formation of the FMN binding pocket (~20 × 9 × 9 Å^3^). Various interactions involved in the fixation of the co-factor FMN in the binding site are summarized in Table 2. The isoalloxazine ring of FMN was stabilized with loops Asp95–Gly103 and Thr59–Asp63, in which Trp60 and Tyr98 located at both sides of the isoalloxazine ring are the most important for providing direct π-π interactions (Figure 3b). The distances from Trp60 and Tyr98 to FMN are 3.9 and 3.7 Å, respectively. The amino acids from Ser10 to Thr15 form a loop that provides several hydrogen bonds to stabilize the phosphate group of FMN.

### 2.4. The Dynamic Characteristics in Fld

**Figure 4:**
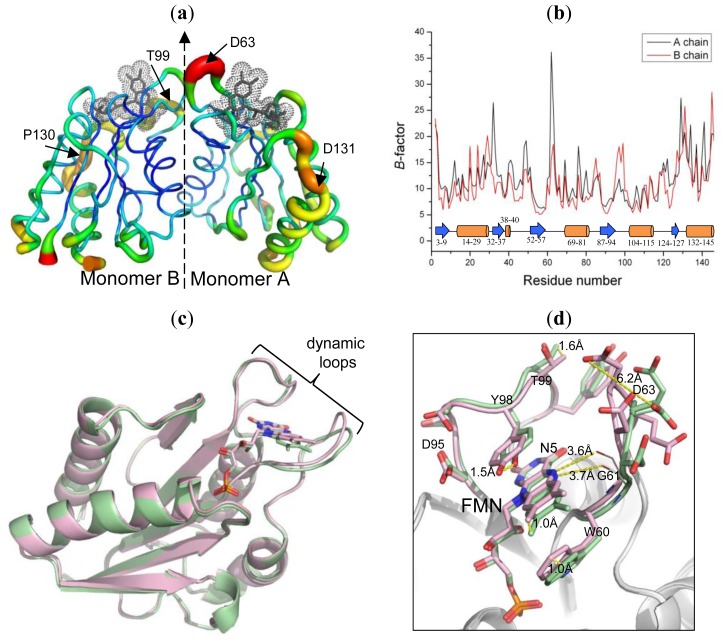
Protein dynamics. (**a**) Temperature-factor puttied structure. The dimer interface was indicated with the dashed line. The residues with large vibrations are labeled. The dot surface represents the structure of FMN located in the binding site; (**b**) The average temperature *B*-values of the dimer structure. The carton diagram shown below represents the secondary structures. Blue: β-sheet; orange: β-helix; (**c**) Superimposed the monomer structures of the Fld dimer. Green: monomer A; pink: monomer B. Two loops with the dynamic feature are labeled; (**d**) An enlarged view of the dynamic loops shows the displacements of residues.

An overall structural flexibility of dimeric Fld was examined with temperature *B*-factors of the residues (Figure 4a). The most dynamic region is the loop Thr59–Asp63 with an average *B*-value ~37 Å^2^ in the monomer A (Figure 4b). The loop Ser96–Thr99 in the monomer B also has a large overall *B*-value (~20 Å^2^). These two dynamic loops are, notably, utilized to coordinate with the co-factor FMN. Moreover, the C-terminal β-helices (Ser132–Arg145) in both monomers also have large *B*-values (~22 Å^2^). The individual residues Glu32 (~27 Å^2^) and Gly49 (~20 Å^2^) in the monomer A show large temperature factors for their dynamic character.

Superimposing the structures of the two monomers in the Fld dimer reveals a small r.m.s. deviation (0.37 Å), implying that the overall folding of each of the monomers is similar (Figure 4c,d), but a detailed inspection of the conformation shows that the structures and positions of some main chains and the co-factors are notably altered, especially a conformational alteration around the active site. This comparison showed that two FMN-coordination loops (Thr59–Asp63 and Ser96–Thr99) are not at the same corresponding position in the two monomers, in which Asp63 and Tyr98 exhibit the largest vibration distances ~6.2 and 1.5 Å, respectively. The position of FMN also exhibits a great deviation, up to 1.0 Å. Moreover, the conformations of residues on the protein surface, such as Glu20, Asp40 and Glu129, are altered.

The structures of the Flds in varied oxidation states were solved in various species, such as *Megasphaera elsdenii* [32], *Clostridium beiherinckii* MP [33], *Anacystis nidulans* [14] and *Desulfovibrio vulgaris* [27]. All structural results indicate that the Flds prefer the reduction from the oxidized state to the semiquinone state, which is accomplished by a peptide flip at the glycine residue (Gly61 in *D. gigas*) and with formation of a hydrogen bond between atom N5 of FMN and the carbonyl oxygen of the glycine main chain (Figure 4d). A similar state was evaluated in the Fld from *D. gigas*, which has the same structural characteristic as a semiquinone state. The peptide of Gly61 points to atom N5 of FMN with distances 3.7 and 3.6 Å, respectively, in monomers A and B. However, the structure and character of FMN are easily affected by X-ray radiation damage [34,35]. Therefore, we could not exclude the possibility that the peptide flipping of Gly61 is raised by the radiation damage.

Several residues of Fld from *D. vulgaris* were investigated by mutations for their roles in modulated redox potentials [29,30]. Superimposed structures of two monomers from the *D. gigas* Fld dimer reveal that those corresponding residues are deviated with different orientations, implying that a similar modulation of the redox potential might exist in *D. gigas* Fld. The side-chain conformations of the conserved residues Gly61 and Asp95 among various Flds from *Desulfovibrio* sp. are altered about 1.0 and 0.7 Å, respectively (Figure S1). In addition, the mutation S64C on the non-conserved residue Ser64 in Fld from *D. vulgaris* might play a role in the redox potential [36]. In *D. gigas* Fld, the corresponding residues Glu64 point their side chains in opposite directions with a deviated distance 5.4 Å, observed in the two superimposed monomer structures.

### 2.5. The Dimerization and Crystal Packing of Fld

**Figure 5:**
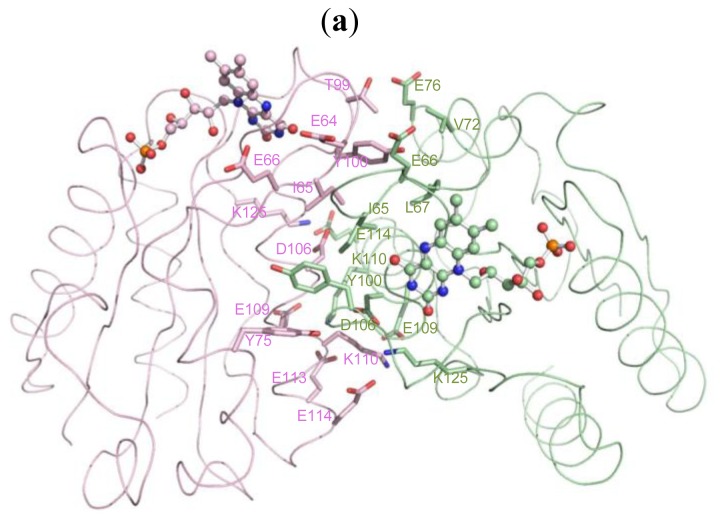

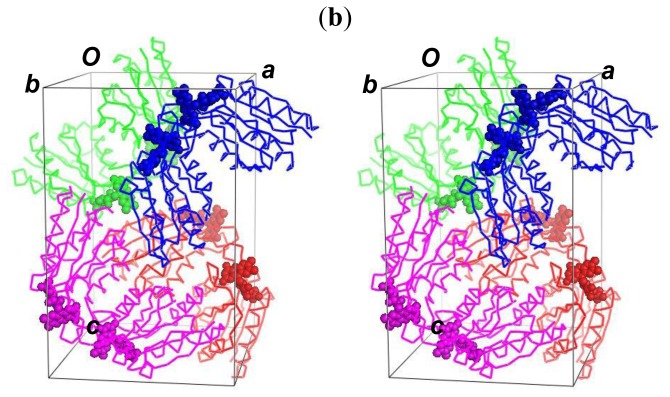
The interface of dimerization and crystal packing. (**a**) Several residues involved in the dimerization of Fld are labeled, which provide major hydrogen bonds with distances within 3.5 Å between the monomers A (green) and B (pink). The FMN is shown in ball and stick; (**b**) The stereo view of the molecular packing in Fld crystals. The co-factor FMN is shown with the sphere model. Each dimer form of Fld is shown with the same color.

The self-rotation function confirms the presence of two-fold axes for the non-crystallographic symmetry, which generates the dimer structure of Fld. If the position of FMN in the Fld is defined as the “head” of the structure, the dimer model is packed in an orientation head to head. The molecular packing in the unit cell shows that there are four dimer structures present in space group *P*2_1_2_1_2_1_ in the crystals (Figure 5b). Gel filtration during protein purification showed that Fld exists as a dimer in solution (Figure S2), which corresponds to the crystal state, implying that the dimeric structure head to head might be the functional unit.

The contact area in the dimeric interface is ~770 Å^2^ (Figure 5a). There are 35 major hydrogen bonds with distances less than 3.5 Å to contribute to this contact area. The residues with interaction distances stabilizing the dimer structure are listed in Table 3. Briefly, two segments are mainly involved in dimerization; one is the dynamic loop Thr59–Asp63 mentioned previously, and the other is the β-helix Gly103–Leu155. The interface of dimerization notably contains no charge or slight positive charge distribution; dimerization hence exposes and creates a more negatively charged environment on the surface of the Fld dimer by concealing the uncharged or positively charged residues at the dimeric interface.

Flds are reported to be multiple functional enzymes, which could interact with various partners, in various reactions [2–12], but the structures of Fld in a complex with its partners are seldom investigated. How the Fld utilizes its key residues to recognize and interact with partner proteins is unclear. Some authors proposed that the displacements of the Trp60-containing loop might be an initial point for the protein-partner recognition [28]. The same loop for the partner recognition was proposed in Fld from *E. coli* [37].

Our Fld structure is a dimer, as in the native state, which allows us to probe the binding site for its partners. From the view of the active site, FMN is the center of the electron transfer in Fld. An inspection of the co-factor environment shows that dimerization could bring two FMN close to each other with a short distance 17 Å. Within the range between two FMN, a large negatively charged region is formed (Figure 2b), which is suitable for the interaction with its protein partner containing the positively charged surface. In addition, two dynamic loops, which might be involved in the protein function mentioned previously, and two FMN from the dimer are positioned in a line with order Thr59–Asp63/A, FMN/A, Asp95–Tyr100/A, Asp95–Tyr100/B, FMN/B and Thr59–Asp63/B (Figure 2b). The loop Thr59–Asp63 is the corresponding loop of the Trp60-contaning loop that was investigated previously [28,37]. The crystal structure of Fld from *D. gigas* might thus explain that the dimeric form is the functional unit due to the aligned negatively charged region formed by dimerization, in which two flexible loops might serve for the protein-partner recognition. Moreover, through dimerization, the isoalloxazine rings of FMN move nearer each other, which could presumably facilitate the transfer of electrons from the electron donor to the acceptor. The dimerization hence likely enables Fld to receive or to donate electrons efficiently from or to the protein partner.

### 2.6. Comparison with Fld Structures from *Desulfovibrio* sp

The structure of Fld from *D. gigas* was compared with the structures from *D. vulgaris* (PDB: 5FX2) and *D. desulfuricans* (PDB: 3KAQ) of a semiquinone state. Superimposed structures of Flds from *D. gigas* with *D. vulgaris* and *D. desulfuricans* reveal a similar folding with the r.m.s.d ~0.55 Å and 0.75 Å, respectively, for all atoms. The major structural differences arise at two dynamic loops Thr59–Asp63 and Asp95–Tyr100, which interact with FMN, and the loop Asp127–Asp131 that contributes to the formation of a FMN-binding pocket. An analysis of the hydrogen bonds between the FMN and residues of Flds from various species shows that the interactions (43) within distance 3.5 Å in *D. gigas* are fewer than those in *D. vulgaris* (51) and *D. desulfuricans* (50), indicating that the binding force of FMN at the active site is weaker in *D. gigas* Fld. The interaction distances between atom N5 of FMN and the carbonyl oxygen of Gly61 in the Fld dimer of *D. gigas* (3.6–3.7 Å) are notably greater than that of *D. vulgaris* (3.1 Å) and *D. desulfuricans* (2.8 Å).

## 3. Experimental Section

### 3.1. Protein Purification

*D. gigas* (ATCC 19364) was anaerobically grown as described previously [38]. All purification steps of Fld were modified from the previous report [39] and performed at 4 °C in a cool room. The cells were suspended in Tris buffer (20 mM, pH 7.6) at concentration about 0.5 g of cells per 1 mL of buffer, subsequently subjected to cell disruption with ultra-sonication and directed through a high-pressure homogenizer (20,000 psi, 2 cycles, EmulsiFlex-C3, Avetin Inc., Ottawa, Canada). The lysed mixture was first centrifuged (12,100× *g*) for 20 min to remove larger particles; the supernatant was collected and ultra-centrifuged (194,000× *g*) for 2 h to remove the membrane fractions. To maintain the sample in the same condition, the supernatant was dialyzed against Tris buffer (20 mM, pH 7.6) over night in a cool room using a dialysis tube (5 kDa M.W. cut-off, purchased form Spectra). The sample was then directly loaded into the first DE-52 column, which was pre-equilibrated with the initial buffer (Tris, 20 mM, pH 7.6). The gradients of Tris (pH 7.6) from 20 mM to 0.5 M served to separate several fractions. The peak eluted around 0.35 M Tris with a yellow color containing Fld was collected and loaded into the second “hydroxyaptite” (HTP) column. After the sample was loaded on the column, the gradient of Tris was decreased from 0.35 M to 0 M; the buffer was then replaced with phosphate (pH 7.6) with gradient increasing from 0 M to 0.4 M. The fractions of the yellow color were collected again and concentrated to load into the third column–Sephadex G50, in which the Fld was purified and desalted. At this stage, SDS-page showed that the Fld factions were still contaminated with other proteins at small fractions. Further purification with the ion-exchange column was thus applied to remove the minor contaminations. The sample was loaded onto the final column—the DEAE Bio-gel, and eluted with a linear gradient of Tris buffer (pH 7.6) from 0.25 M to 0.5 M. The yield of the protein was approximately 0.2 mg per gram of *D. gigas* cells; the pure protein was analyzed with UV-visible spectra and SDS-PAGE (12%) with Coomassie Brilliant Blue staining.

### 3.2. Crystallization

Before crystallization trials, the protein sample was centrifuged to a concentration 8 mg/mL in Tris buffer (20 mM, pH 7.6). The crystal was screened in 96-well VDX^™^ plates with the crystallization robot (Thermo Scientific Matrix Hydra II eDrop) according to the sitting-drop vapor-diffusion method at 18 °C. The crystallization plates were stored and monitored (Rocker Imager 54 (Formulatrix). Small crystals were observed from a condition containing PEG550 (30%, *v*/*v*), calcium chloride dihydrate (50 mM) and bis-Tris buffer (100 mM, pH 6.5) within six days after the initial screening using the Index kit (Hampton Research Co., Aliso Viejo, CA, USA). This condition was further refined to produce Fld crystals a little larger on varying the pH of the buffers and concentrations of PEG550 and CaCl_2_; the improvement of the crystal quality was, however, inadequate for satisfactorily X-ray diffraction. To obtain crystals of superior quality, we performed the micro-seeding technique. The small crystals (0.1 × 0.1 × 0.1 mm) were first crushed into microcrystals with a thin glass wand in the Eppendorf (500 μL) containing the mother liquid solution, and transferred to fresh mother liquid for washing and dilution, several times. Only a few microcrystals were finally transferred to the hanging drops (2 μL) containing protein solution (1 μL) and reservoir solution (1 μL) in equal volumes, against the bottom reservoir solution (500 μL) containing PEG550 (25%, *v*/*v*), calcium chloride dihydrate (50 mM) and bis–Tris buffer (100 mM, pH 6.5) in the 24-well plate. Single crystals were observable two days after the micro-seeding technique and hang-drop vapor-diffusion method. Improved crystals of quality satisfactory for X-ray diffraction were used for data collection. The details of the data statistics are given in Table 1.

### 3.3. X-ray Data Collection and Processing

The protein crystals were initially screened and characterized using synchrotron radiation as a source of X-ray at SPXF beamline BL13B1 equipped with a CCD detector (Q315, ADSC, Poway, CA, USA) at National Synchrotron Radiation Research Center (NSRRC, Taiwan). Data collection was completed at Taiwan-contracted protein crystallographic beamline BL12B2 equipped with a CCD detector (Quantum-4R, ADSC, Poway, CA, USA) at SPring-8 in Japan. The crystal was transferred from a crystallization drop into a cryo-protectant solution (10 μL) containing PEG550 (25%, *v*/*v*), calcium chloride dihydrate (50 mM), glycerol (20%, *v*/*v*), and bis-Tris buffer (100 mM, pH 6.5) for a few seconds and mounted on a synthetic nylon loop (0.4–0.5 mm, Hampton Research Co., Aliso Viejo, CA, USA), and then flash-cooled in liquid nitrogen. For complete data collection, 180° rotations with 1.0° oscillation were measured with X-ray wavelength of 1.00 Å, exposure duration 15 s and distance 150 mm from the crystal to the detector, at 110 K in a dinitrogen stream using a cryo-system (X-Stream, Rigaku/MSC, Inc., Tokyo, Japan). All data were indexed, integrated and scaled using programs *HKL2000* [40].

### 3.4. Structural Determination and Refinement

The crystal structure of Fld was solved by molecular replacement using the structure of Fld from *D. vulgaris* (57.4% sequence identity; PDB code 1FX1 [27]) (Figure S1) as a search model. A molecular replacement solution was obtained with a correlation coefficient of 0.406 (the next highest solution had correlation coefficient 0.230) in the resolution range 20–4.0 Å using *CNS v.*1.2 [41], which confirmed the presence of two protein molecules per asymmetric unit. A randomly selected 5% of total observed reflections served as a test set for the free *R*-factor calculation. The model was rebuilt and adjusted according to the electron density with program *Coot* [42]. After rigid-body refinement using *CNS v.1.2* [39] in the resolution range 30–3 Å, factors *R* = 45.9% and *R*_free_ = 47.9% were obtained. After several cycles of manual adjustment and refinement, multiple conformations of various amino-acid residues, the model was refined with *REFMAC5* [43], which generated an *R* factor about 25%. The water molecules were added with *water_pick* in *CNS v.1.2* [44], which generated about 380 water molecules. After inspections of the electron density, 34 water molecules were deleted because of the low electron density level, generating the final model with factors *R* = 18% and *R*_free_ = 21%. The final structure was evaluated with *RAMPAGE* [45] and *PROCHECK* [43]. All figures of structures and electron densities were generated with *PyMOL* (http://www.rcsb.org) [46]. Coordinate and structure factor of Fld from *D. gigas* have been deposited with PDB under the accession code 4HEQ.

## 4. Conclusions

We isolated, purified and crystallized the “short-chain” Fld from *D. gigas* for structural investigation. The results show that the structure of Fld from *D. gigas* is a dimer containing two FMN molecules with the monomers orientated head to head, which agrees with the solution state. The dimerization formed a long and connected, negatively charged, surface that is suitable for interaction with its electron-transferring partners. The loops coordinating FMN are dynamic and located in the dimerization interface, implying that the loops might assist the binding of electron partners.

## Supplementary Information



## Figures and Tables

**Table 1:** Data collection and refinement statistics.

Data collection	
Wavelength (Å)	1.00
Temperature (K)	110
Space group	*P*2_1_2_1_2_1_
Resolution Range (Å)	30.0–1.21 (1.25–1.21) a
Cell dimensions (Å)	
*a*	50.20
*b*	60.37
*c*	76.25
Unique reflections	71,508 (7049) a
Completeness (%)	99.9 (99.7) a
*<I/σ*(*I*)*>*	33.9 (3.4) a
Average redundancy	7.1 (6.8) a
*R*_sym_^b^ (%)	8.7 (71.8%) a
Mosaicity	0.28
No. of molecules per asymmetric unit	2
Matthews coefficient (Å^3^ Da^−1^)	2.06
Solvent content (%)	40.4

**Refinement**	

Resolution range (Å)	30.0–1.3
*R*^workc^/*R*^freed^ (%)	18.0/21.1
No. of atoms	
Protein	2152
Ligand (FMN)	61
Water molecules	346
*B*-factors (Å^2^)	
Protein	11.5
Ligand (FMN)	8.0
Water molecules	21.1
R.m.s deviations	
Bond lengths (Å)	0.027
Bond angles (°)	2.460

a Values in parentheses are for the highest resolution shell (1.36–1.3 Å);

b *R*_sym_ = ∑*_h_* ∑*_i_* [|*I_i_*(*h*) − <*I*(*h*)>|/∑*_h_* ∑*_i_I_i_*(*h*)], where *I_i_* is the *i*th measurement and <*I*(*h*)> is the weighted mean of all measurements of *I*(*h*);

c *R*_work_ = ∑h|*F*o *− F*c |/∑h *F*o, where *F*o and *F*c are the observed and calculated structure factor amplitudes of reflection h.

d *R*_free_ is as *R*_work_, but calculated with 10% of randomly chosen reflections omitted from refinement.

**Table 2:** The interactions between FMN and surrounding residues.

FMN	Contact	Atoms	Distance (Å) (monomer A)	Distance (Å) (monomer B)
O3P [O]	12(THR)	N [N]	2.93	2.88
	14(ASN)	N [N]	2.93	2.92
	12(THR)	OG1 [O]	2.56	2.57

O1P [O]	60(TRP)	NE1[N]	2.82	3.2
	58(SER)	OG [O]	2.74	2.71
	11(THR)	N [N]	2.82	2.78

O2P [O]	15(THR)	N [N]	2.73	2.71
	15(THR)	OG1 [O]	2.75	2.76
	10(SER)	OG [O]	2.71	2.69

O4′ [O]	14(ASN)	ND2 [N]	2.84	2.86

O2′ [O]	59(THR)	O [O]	2.72	2.7

O2 [O]	95(ASP)	N [N]	2.94	2.91
	102(CYS)	N [N]	2.78	2.78

**Table 3:** The residue contacts between the dimer interface.

Source (chain/residue)	Atoms	Target (chain/residue)	Atoms	Distance (Å)
A/64(GLU)	CB [C]	B/65(ILE)	O [O]	3.30

A/65(ILE)	N [N]	B/65(ILE)	O [O]	3.20

A 65(ILE)	O [O]	B/64(GLU)	CA [C]	3.35
		B/65(ILE)	N [N]	2.82

A/66(GLU)	CG [C]	B/63(ASP)	O [O]	3.45

A/66(GLU)	CD [C]	B/63(ASP)	O [O]	3.35

A/67(LEU)	N [N]	B/100(TYR)	OH [O]	3.03

A/72(VAL)	CG2 [C]	B/100(TYR)	OH [O]	3.49

A/76(GLU)	OE2 [O]	B/99(THR)	CG2 [C]	3.44
		B/99(THR)	OG1 [O]	2.62

A/100(TYR)	CD1 [C]	B/75(TYR)	CE2 [C]	3.49

A/106(ASP)	OD1 [O]	B/110(LYS)	CG [C]	3.16

A/109(GLU)	OE1 [O]	B/110(LYS)	CE [C]	3.33
		B/110(LYS)	NZ [N]	2.87

A/110(LYS)	CB [C]	B/106(ASP)	OD1 [O]	3.40

A/110(LYS)	CD [C]	B/109(GLU)	OE1 [O]	3.18

A/110(LYS)	CE [C]	B/109(GLU)	OE1 [O]	3.12

A/110(LYS)	NZ [N]	B/109(GLU)	CD [C]	3.45
		B/109(GLU)	OE1 [O]	2.74
		B/113(GLU)	CD [C]	3.25
		B/113(GLU)	OE1 [O]	2.60
		B/113(GLU)	OE2 [O]	3.20

A/114(GLU)	OE2 [O]	B/125(LYS)	NZ [N]	2.85

A/125(LYS)	NZ [N]	B/114(GLU)	OE2 [O]	2.83
