# Supplementary material for: Crystal Structure of Dimeric Flavodoxin from Desulfovibrio gigas Suggests a Potential Binding Region for the Electron-Transferring Partner
Source: Int J Mol Sci. 2013 Jan 15;14(1):1667–83. doi: 10.3390/ijms14011667 (PMC3565340; doi:10.3390/ijms14011667)
Supplement: Supplementary file 1 [file ijms-14-01667-s001.pdf]

Supplementary Information

Figure S1. The sequence alignment of selected *Desulfovibrio sp*. The blue and black boxes indicate the residues involved in the negatively charged surface and the dimeric interface, respectively.

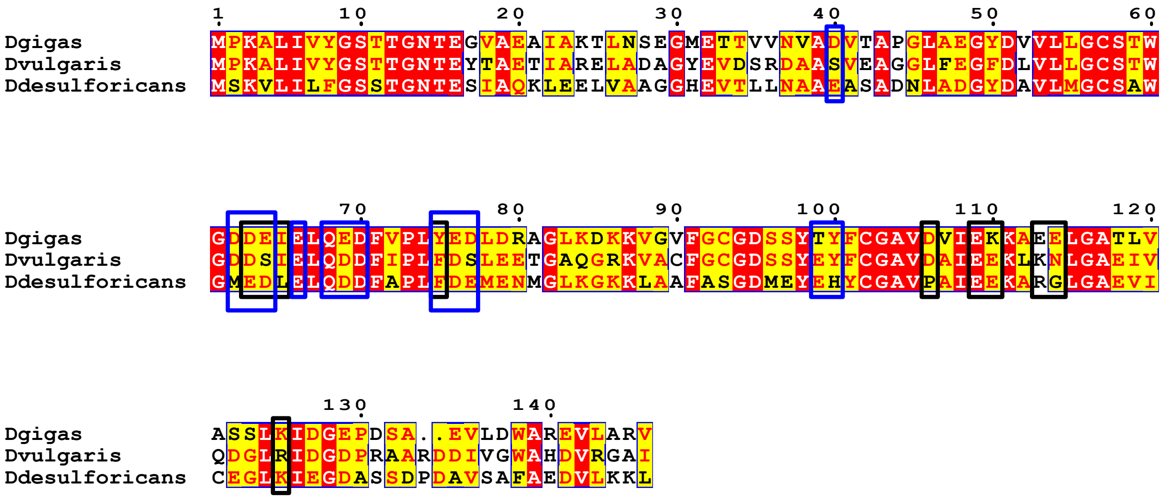

Figure S2. The calculation of the molecular weight by gel filtration. The calculated molecular weight of Fld is ~26 kDa by gel filtration through superdex-G75, which is the twice monomeric molecular weight (15 kDa). Standard materials: Aldolase (158 kDa), Ovalbumin (44 kDa), Ribonuclease (13.7 kDa), Aprotinin (6 kDa).

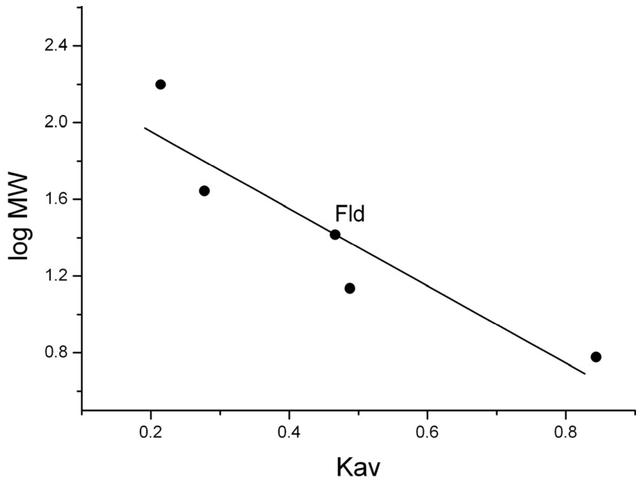

© 2013 by the authors; licensee MDPI, Basel, Switzerland. This article is an open access article distributed under the terms and conditions of the Creative Commons Attribution license (http://creativecommons.org/licenses/by/3.0/).
